# CTC together with Shh and Nrf2 are prospective diagnostic markers for HNSCC

**DOI:** 10.1186/s12860-024-00500-0

**Published:** 2024-02-10

**Authors:** Md. Mizanur Rahman, Muhammad Mosaraf Hossain, Shafiqul Islam, Ridwan Ahmed, Mohit Majumder, Shantu Dey, Md. Kawser, Bishu Sarkar, Md. Ejajur Rahman Himu, Ali Asgar Chowdhury, Shakera Ahmed, Supran Biswas, Mostafa Mahfuzul Anwar, Mohammad Jamal Hussain, Rajib Kumar Shil, Sunanda Baidya, Ramendu Parial, Mohammed Moinul Islam, Atul Bharde, Sreeja Jayant, Gourishankar Aland, Jayant Khandare, Shaikh Bokhtear Uddin, Abu Shadat Mohammod Noman

**Affiliations:** 1 Rangamati Medical College, Rangamati, Bangladesh; 2 Department of Biochemistry & Molecular Biology, University of Chittagong, Chattogram, 4331 Bangladesh; 3 EuGEF Research Foundation, Chattogram, Bangladesh; 4 Department of Radiotherapy, Chittagong Medical College, Chattogram, Bangladesh; 5 Department of Surgery, Chittagong Medical College, Chattogram, Bangladesh; 6 Department of Otolaryngology and Head Neck Surgery, Chittagong Medical College, Chattogram, Bangladesh; 7 Department of Otolaryngology and Head Neck Surgery, Rangamati Medical College, Rangamati, Bangladesh; 8 Actorious Innovations and Research Pvt. Ltd., India and Simi Valley, Pune, CA USA; 9 Department of Botany, University of Chittagong, Chattogram, Bangladesh; 10 Present Address: Stem Cell Genetics, Institute of Life and Medical Sciences, Kyoto University, Kyoto, Japan

**Keywords:** CTC, Shh, Nrf2, HNSCC, Cancer

## Abstract

**Background:**

The lack of appropriate prognostic biomarkers remains a significant obstacle in the early detection of Head and Neck Squamous Cell Carcinoma (HNSCC), a cancer type with a high mortality rate. Despite considerable advancements in treatment, the success in diagnosing HNSCC at an early stage still needs to be improved. Nuclear factor erythroid 2-related factor 2 (Nrf2) and Sonic Hedgehog (Shh) are overexpressed in various cancers, including HNSCC, and have recently been proposed as possible therapeutic targets for HNSCC. Circulating Tumor Cell (CTC) is a novel concept used for the early detection of cancers, and studies have suggested that a higher CTC count is associated with the aggressiveness of HNSCC and poor survival rates. Therefore, we aimed to establish molecular markers for the early diagnosis of HNSCC considering Shh/Nrf2 overexpression in the background. In addition, the relation between Shh/Nrf2 and CTCs is still unexplored in HNSCC patients.

**Methods:**

In the present study, we selected a cohort of 151 HNSCC patients and categorized them as CTC positive or negative based on the presence or absence of CTCs in their peripheral blood. Data on demographic and clinicopathological features with the survival of the patients were analyzed to select the patient cohort to study Shh/Nrf2 expression. Shh and Nrf2 expression was measured by qRT-PCR.

**Results:**

Considering significant demographic [smoking, betel leaf (*p*-value < 0.0001)] and clinicopathological risk factors [RBC count (*p* < 0.05), Platelet count (*p* < 0.05), Neutrophil count (*p* < 0.005), MCV (*p* < 0.0001), NLR (*p* < 0.05), MLR (*p* < 0.05)], patients who tested positive for CTC also exhibited significant overexpression of Shh/Nrf2 in both blood and tissue compared to CTC-negative patients. A strong association exists between CTCs and tumor grade. Following chemotherapy (a combination of Cisplatin, 5FU, and Paclitaxel), the frequency of CTCs was significantly decreased in patients with HNSCC who had tested positive for CTCs. The Kaplan–Meier plot illustrated that a higher number of CTCs is associated with poorer overall survival (OS) in patients with HNSCC.

**Conclusions:**

Detecting CTCs, and higher expression of Shh and Nrf2 in HNSCC patients’ blood, can be a promising tool for diagnosing and prognosticating HNSCC.

**Supplementary Information:**

The online version contains supplementary material available at 10.1186/s12860-024-00500-0.

## Introduction

Head and neck squamous cell carcinoma (HNSCC) is a cancer that arises from the malignant transformation of the squamous epithelial cells found in the head, neck, and oral cavity. The tumor originates from various anatomical sites shielded by squamous epithelium, including the aero-digestive tract, oral cavity, paranasal regions, nasopharynx, hypopharynx, and larynx [1]. HNSCC ranked as the seventh most prevalent cancer worldwide, with breast cancer being the highest in women and lung cancer in men [2]. In Bangladesh, head and neck cancer is the most common type of cancer in both men and women, followed by lung cancer in men and breast cancer in women [3]. HNSCC is significant due to its five-year low overall survival rate (50%) and the absence of sensitive diagnostic and prognostic biomarkers [4]. Several antibody-based biomarkers, such as CA-125, CEA, PSA, and others, have been developed for detecting various cancers, for example, CA 125 for early detection of ovarian cancer, CEA for colorectal cancer, and PSA for prostate cancer [5]. However, recent studies demonstrated that overexpression of Shh and Nrf2 contributes to the development of HNSCC [6].

Sonic hedgehog (Shh) is dysregulated in head and neck squamous cell carcinomas (HNSCC) [6]. Shh overexpression is associated with metastasis and tumor growth [7], maintenance of cancer stem cells (CSCs) [8], and resistance to chemotherapy [9]. During off state, PTCH1 (a 12-transmembrane protein) inhibits SMO (a 7-transmembrane protein), while SUFU (Suppressor of fused homolog) binds to GLI1 (a downstream regulator of the Shh pathway), preventing transcriptional activation. However, in the active state, when Shh binds to the PTCH1 receptor, inhibition of SMO is lifted, releasing GLI1 from SUFU. GLI1 then enters the nucleus, activating target genes such as PTCH1, CCND1 (cyclin D1), MYC (Master Regulator of Cell Cycle Entry), and others, resulting in proliferation and suppression of apoptosis [10].

Usually, in the cytoplasm, Nrf2 (Nuclear factor erythroid 2-related factor 2 (Nrf2) and Keap1 (Kelch-like ECH-associated protein 1) bind each other and cause proteasomal degradation resulting in antioxidant activity, detoxification, and cancer prevention. In cancer, Nrf2 unbinds from Keap1, enters the nucleus, and causes activation of transcription factors, resulting in drug resistance, enhanced cell proliferation, stress adaptation, and activation of metabolic reprogramming [11]. Interestingly, loss of function of KEAP1 or gain of function of Nrf2 has been reported in HNSCC [12]. In HNSCC, Nrf2 is overexpressed in 90% of cases and found to correlate with poor overall survival [13].

Identification of circulating tumor cells (CTCs) is a liquid biopsy tool [14] approved by the US FDA for identifying and enriching CTCs from the blood of colorectal, prostate, and breast cancer patients [15]. Recent studies have reported the significance of CTC-based diagnosis in various cancers, including HNSCC [16]. In HNSCC patients, the presence of CTCs in the blood is associated with poor disease-free survival [17]. CTC enumeration has proven helpful in metastatic settings for predicting overall survival, monitoring treatment response, and post-treatment surveillance in HNSCC [18]. Cancer cells shed from the site of initiation and enter the circulation through a process known as intravasation. These circulating cells then undergo extravasation and migration at distant sites, potentially leading to distal metastasis [19]. Despite the advancements in chemotherapy and radiotherapy, the overall 5-year survival rate for early and advanced HNSCC remains approximately 50%, with 20%, respectively. The number of CTCs correlates with the aggressiveness of HNSCC metastasis and poorer survival rates [20]. In HNSCC, the main challenges lie in early metastasis detection and prompt treatment initiation. Therefore, overexpression of Shh and Nrf2 and detection of CTCs could serve as promising prognostic and diagnostic biomarkers for HNSCC. Moreover, the relationship between Shh/Nrf2 and CTCs has yet to be explored as a potential biomarker for HNSCC patients. The present study aimed to establish an effective diagnostic and prognostic tool for the early detection of metastasis by linking biomarkers (Shh/Nrf2) with CTCs in HNSCC patients. Our findings revealed that Shh/Nrf2 expression was upregulated in the blood and tissues of CTC-positive patients compared to CTC-negative patients. For HNSCC patients who tested positive for Shh/Nrf2 expression with CTCs, the frequency of CTCs significantly decreased at the post-treatment state. Furthermore, patients with HNSCC who had a higher number of CTCs exhibited a worse overall prognosis. In conclusion, the combination of Shh/Nrf2 overexpression and CTC detection can be a potential tool to predict early diagnosis and prognosis in HNSCC patients.

## Method and materials

### Place and duration of the study

This cross-sectional prospective study on Head and Neck Squamous Cell Carcinoma (HNSCC) patients was conducted between February 20, 2020, and September 8, 2022, at the Department of Otolaryngology and Head Neck Surgery, Chattogram Medical College Hospital (CMCH), Bangladesh and the fundamental bench work was accomplished at the Centre for Research Excellence (CRE), Department of Biochemistry and Molecular Biology, University of Chittagong, Bangladesh. The CTC detection and analysis was performed at Actorius Innovations and Research Pvt Ltd, Pune, Maharashtra, India.

### Sample size determination

Based on GLOBOCAN’s [3] estimate of the prevalence rate of HNSCC in Bangladesh (~ 7–8%), we included 151 HNSCC patients who met the study’s inclusion criteria, which included being over the age of 18, being willing to participate, and not having any concurrent medical conditions like diabetes, hypertension. A certified pathologist carried out the histopathological examination of tumor tissue sections to confirm the diagnosis of cancer. We excluded patients who refused to take part in our study. Peripheral blood was drawn from the study participants to measure the expression of Nrf2 and Shh and to identify Circulating Tumor Cells (CTCs). To compare the Shh and Nrf2 expression with the HNSCC patients, 24 healthy controls blood samples were examined. Shh and Nrf2 gene expression were also measured using tumor tissue samples of the study subjects.

### Data analysis from TCGA

To get a glimpse into the expression pattern of Shh and Nrf2, cBioPortal and the UALCAN online computational tools were used to analyze Shh and Nrf2 RNA Sequence data of 523 patients from the TCGA (The Cancer Genome Atlas) dataset. Transcript per million (TPM) in the TCGA dataset was used to determine the level of Shh and Nrf2 gene expression profile (TPM < 1). Shh and Nrf2’s mRNA expression patterns were determined from 515 expressed genes, and the data were verified on all platforms using the ± 1 z-score threshold as a cutoff to identify high and low levels of Shh and Nrf2.

### Blood sample collection for identification of CTCs and gene expression study

Peripheral blood was collected from the study subjects in cooperation with the Department of Otolaryngology and Head Neck Surgery (ENT), Chattogram Medical College and Hospital, Chittagong. A certified phlebotomist was assigned to draw and collect the HNSCC patient’s blood. A total of 4.5 ml peripheral blood was drawn from each study subject, of which 1.5 ml was used to detect CTCs using the OncoDiscover platform mediated by anti-EpCAM antibody conjugated affinity-based magnetic nanoparticles approved by the Drug Controller General of India. The rest of the blood was utilized for gene expression study.

### Identification of CTCs in blood samples of HNSCC patients

An aliquot of 1.5 ml blood was used for CTC detection by the OncoDiscover platform. Briefly, the Oncodiscover CTC testing platform comprises the following constituents (i) anti-EpCAM (Anti Epithelial Cell Adhesion Molecule Antibody) (Bioss, USA), (ii) crosslinked to iron oxide (Fe3O4) nanoparticles, (iii) PEG, (iv) multiwall carbon nanotube, (v) poly (N-isopropyl acrylamide) (PNIPAM) (vii) glutathione (GSH), and (vii) fourth generation (G4) dendrimers with 64 binding sites (European and Indian Patents, 3259598, 384104 respectively). Magnetic nanoparticles attached with anti-EpCAM antibody specified to target cancer cells of epithelial origin directed to capture CTCs. The CTCs were captured by applying a magnetic field, followed by separation and processing by immunofluorescence. Finally, CTCs were confirmed using positive staining of CK 18 (cytokeratin 18) (Abcam, USA) (Cytokeratin expressed in epithelial cells), DAPI (Sigma, USA) (for nucleus), and the absence of CD45 (Bioss, USA) signal (for leukocytes).

### Assembly of demographics, socioeconomic and clinicopathological data

As previously indicated, we divided our study cohort into two groups—CTC positive and CTC negative—based on the presence of CTCs (circulating tumor cells) in the patient’s blood. Demographic, socioeconomic, and clinicopathological findings were considered to narrow down our targeted gene analysis. A clearly defined questionnaire was used to list and store all this data.

### Collection of tissue samples for gene expression study

During surgery, HNSCC tumor tissue was obtained from patients whose cancer had been previously diagnosed by skilled Pathologists. With all aseptic measures and proper storage conditions, tumor tissue samples were preserved in RNAlater (ThermoFisher Scientific) for RNA isolation, followed by measuring the expression of Shh and Nrf2 by qRT-PCR.

### Estimation of hematological parameters

We tested the hematological parameters of our patient cohort to identify correlations between the biomolecules. Red blood cell (RBC) was counted in million/cmm using the Neubauer Chamber method. White blood cell (WBC) count was conducted in thousand/ml using the Neubauer Chamber method. Lymphocyte, Eosinophil, Monocyte, Neutrophil, and Platelet counts were determined using the Leishman staining method. Hematocrit was manually calculated using the formula HCT = (RBC x MCV)/10. Hemoglobin levels were measured using photoelectric colorimetry. Mean corpuscular hemoglobin (MCH) was calculated manually by multiplying the percent hematocrit by ten and dividing it by the erythrocyte count. Serum Creatinine levels were measured using the Jaffe method. Mean corpuscular hemoglobin concentration (MCHC) was calculated manually by multiplying the hemoglobin result from the complete blood count (CBC) panel by 100 and then dividing it by the hematocrit result. Serum Glutamic Pyruvic Transaminase (SGPT) levels were determined using a colorimetric method, and erythrocyte sedimentation rate (ESR) was measured using the Wintergreen method. Mean corpuscular volume (MCV) levels were measured according to physiological protocol by multiplying the hematocrit by ten and dividing it by the RBC count. Neutrophil-to-lymphocyte ratio (NLR) and monocyte-to-lymphocyte ratio (MLR) levels were calculated manually.

### RNA isolation from HNSCC patient’s blood and tissue samples

The chloroform-isopropanol method was used to isolate RNA from blood. In brief, an aliquot of 500 µL of RBC lysis buffer was added per 1 ml sample in the tube. After a gentle mixing, the mixture was let sit for five minutes at room temperature. The tube was then centrifuged for 12 min at 4ºC at 2500 rpm (600 g). Once the supernatant was disposed of, 200–300 µL of RBC lysis buffer was added and carefully blended. The tube was centrifuged for eight minutes at 4ºC and 2500 rpm. This process was repeated until the pellet turned white. After washing the cell pellet in PBS, the tube was chilled to -20ºC. The next day, the tube was vortexed with 800 µL of TRIzol (Life Technologies, USA) added to it. Following that, 200 µL of chloroform was added and vigorously vortexed. After that, the tube was kept on ice for twenty minutes. The tube was then centrifuged for 20 min at 4ºC at 13,000 rpm. The upper aqueous phase was transferred to a new tube. The same volume of isopropanol was added and vortexed, and then the mixture was kept at -20ºC for overnight incubation. It was then put on ice for fifteen minutes. The tube was centrifuged at 13,000 rpm for 20 min at 4ºC the next day. With caution, the supernatant was aspirated. The 500 µL of ice-cold, 75% ethanol washed over the RNA pellets at 4ºC. The RNA pellet was allowed to air dry after the supernatant was carefully drained off. The RNA pellet was then dissolved in the proper volume of RNase-free water. Using a nanodrop spectrophotometer (ND, 2000), the RNA was then quantified and stored at -40ºC for use in subsequent analyses, such as cDNA synthesis, quantitative real-time PCR (qPCR), and agarose gel electrophoresis.

RNA was extracted from the tumor tissue sample using the Chloroform-Trizol method. A tissue sample weighing between 50 and 100 mg was taken and put into the Biomasher II tube. Tissue sample was washed using 1 mL of PBS in the tube. After that, a pestle was placed inside the tube, and 30–400 µL of TRIzol was added. The tissue was disrupted by pressing the pestle against the side of the tube until the sample was fully homogenized. Next, the sample was incubated at room temperature for 2–3 min. Chloroform (half of Trizol volume) was added and inverted the tube carefully. The sample was subsequently kept on ice for twenty minutes. The sample was then centrifuged for 30 min at 4ºC at 13000 rpm. A fresh tube was used to transfer the upper layer. An equal amount of ice-cold isopropanol (99.99%) was added to the tube. After gently inverting the tube, it was set on ice for a duration of 15 to 20 min. Following an overnight incubation period, the samples were centrifuged for 20 min at 4ºC at 13000 rpm. Once the RNA pellet was apparent, the supernatant was cautiously disposed of. The pellet was then washed and allowed to air dry with 500 µL of ice-cold ethanol added. The RNA pellet was then dissolved in the proper volume of RNase-free water. Finally, the concentration of RNA was measured by a nanodrop spectrophotometer (ND, 2000) and kept at -40ºC for subsequent tests, such as cDNA synthesis, agarose gel electrophoresis, and quantitative real-time PCR (qPCR).

### cDNA synthesis and qRT-PCR (Quantitative Real-Time PCR)

Total RNA isolated from blood and tissue samples was used to synthesize cDNA using the GoScriptTM Reverse transcription system sourced from Promega (Cat no: ADM1701 00000654925) per the manufacturer’s protocol. Real-time polymerase chain reaction (PCR) is a commonly used molecular biology technique that amplifies the copy number of a specific gene. The expression levels of the studied genes (Shh, NRF2) were analyzed using real-time quantitative PCR with the GoTaq qPCR master mix (Promega, USA). Working cDNA samples were prepared by diluting the stock cDNA (1 µg/µl) by 100 times. Forward and reverse primers were then added to the samples. Finally, SYBR Green (Cat No: A6000, Promega, USA) was added to each sample tube, thoroughly mixed, and loaded into the RT-PCR machine (BIO-RAD; CFX96TM). The reaction was carried out using specific conditions optimized for the targeted gene. The dsDNA was denatured into single-stranded DNA (ssDNA) by incubating at 95 °C for 3 min. The annealing temperature, which depends on the melting temperature of the primers, was set at 53 °C to allow for the synthesis of new DNA strands from the template. Finally, the extension step was set at 72 °C to facilitate the extension of the new DNA strands from the primers. The relative expression of Shh and Nrf2 was analyzed by the △△CT method, normalizing with GAPDH. The primers are listed in the Supplementary Table 1.

### Statistical analysis

Graph Pad Prism (Version 8.4.2) was chosen as the statistical tool for all statistical analyses. Experiments related to qRT-PCR were replicated three times, and results were presented as mean ± SD. The Mann–Whitney statistical analysis was adopted to compare the tested genes’ expression in CTC-positive and CTC-negative patients. An unpaired t-test was performed to compare hematological parameters between CTC-positive and CTC-negative patients. One-way ANOVA was done to compare the grade of CTC-positive patients and the number of CTCs. Multiple linear regression analysis was done where CTC was a dependent variable against different hematological parameter coefficients. A log-rank (Mantel-Cox) test was performed for survival analysis. The values with a *p*-value < 0.05 were deemed statistically significant.

## Results

### Expression of Shh and Nrf2 in HNSCC in TCGA data cohort

**Fig. 1:**
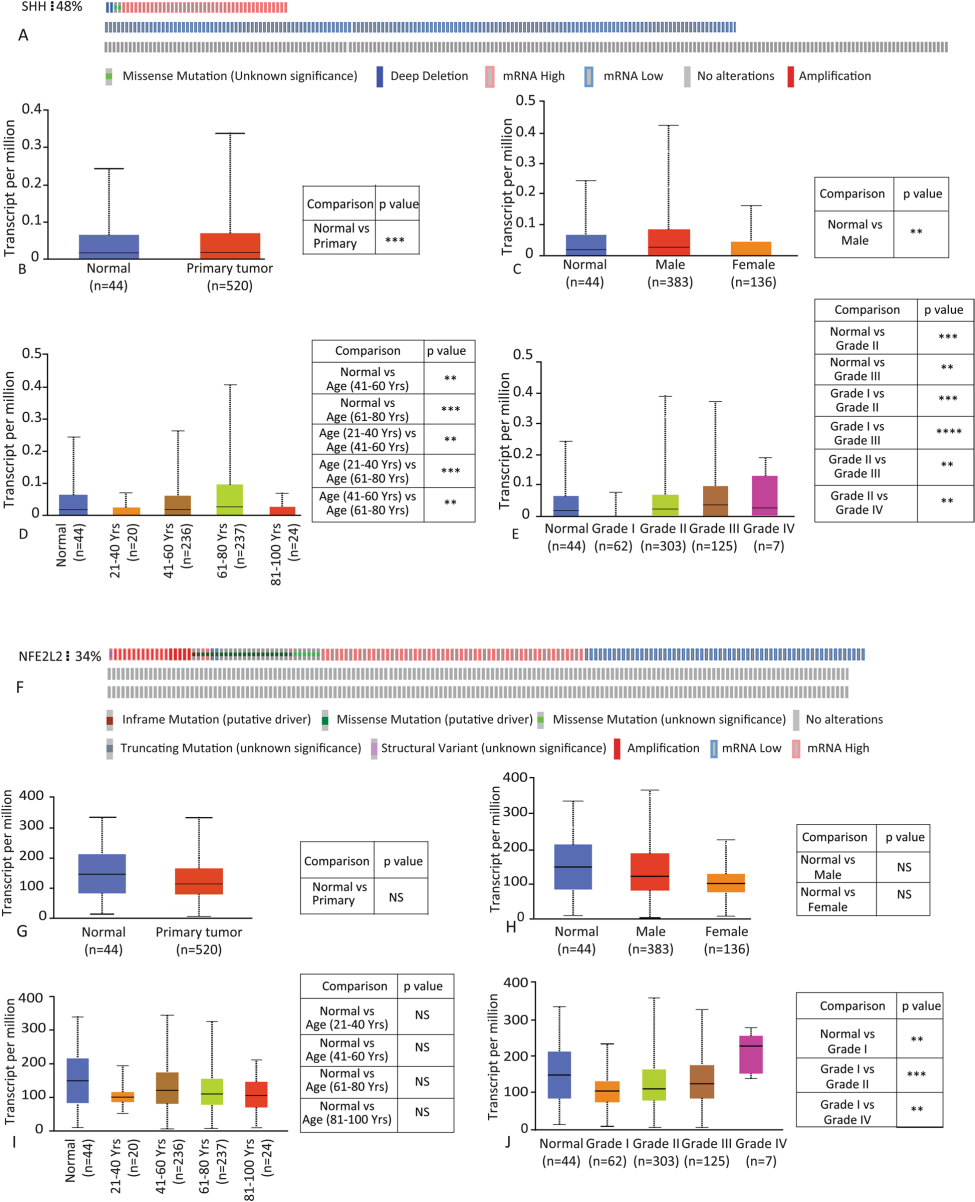
TCGA data analysis for Shh / Nrf2 gene expression in HNSCC patients. **A** The percentage (%) of HNSCC patients showing altered expression of Shh. **B**, **C** Expression of Shh in HNSCC based on sample type and gender. **D**, **E** Expression of Shh in HNSCC patients based on age group and tumor grade. **F** The percentage (%) of HNSCC patients showing altered expression of NFE2L2 (Nrf2). **G**, **H** Expression of Nrf2 in HNSCC based on sample type and gender. **I**, **J** Expression of Nrf2 in HNSCC patients based on age group and tumor grade. Shh and Nrf2 expressions were calculated in the number of transcripts per million. The expression pattern of Shh was checked based on the mRNA Z-score. **p-value < 0.005, ***p-value < 0.001, ****p-value < 0.0001

The TCGA applies high-throughput genome analysis techniques to identify alterations in the DNA and RNA of various tumor types. We examined the TCGA data set to investigate the expression pattern of both the Shh and Nrf2 genes in different tumor types. Based on the mRNA expression in HNSCC, the results indicated that Shh and Nrf2 expression levels were altered in 48% and 34% of HNSCC patients, respectively (Fig. 1A and F). Primary tumors exhibited higher levels of Shh expression than normal tissue, and male HNSCC patients had significantly higher levels than normal individuals (Fig. 1B and C). Compared to normal controls, Shh expression was significantly higher in HNSCC patients of 41–60 and 61–80 age groups and in HNSCC patients of stages 2 and 3 (Fig. 1D and E). On the contrary, no compelling disparity was observed in Nrf2 expression between normal and primary tumor tissue or between males and females (Fig. 1G and H). Nrf2 expression was reduced in patients of different ages compared to normal individuals. However, the reduction was not statistically significant (Fig. 1I). When comparing grade 1 to normal and grade 2 to grade 1, Nrf2 expression was considerably downregulated; however, when comparing grade 4 to grade 1 HNSCC patients, the expression was significantly higher (Fig. 1J). Therefore, Shh and Nrf2 are differentially expressed in HNSCC patients.

### Identification of CTCs from peripheral blood samples of HNSCC patients

**Fig. 2:**
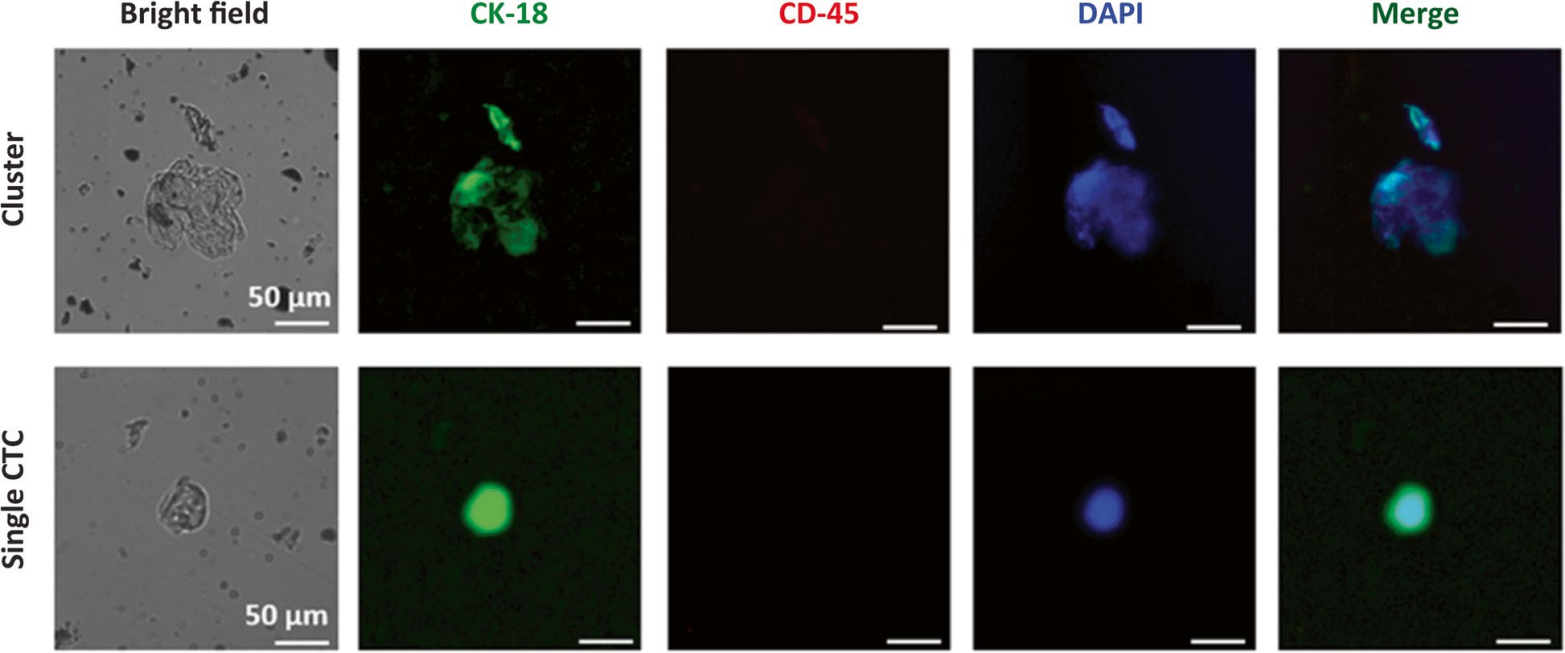
CTC detection from peripheral blood. Images of CTCs detected in HNSCC patients captured by fluorescence microscope where staining with DAPI emits a blue signal, and CK-18 emits a green signal. The top panel represents a cluster of CTCs, and the lower panel represents a single CTC

CTC was detected as a single cell or cluster by fluorescence microscopy analysis of peripheral blood samples from HNSCC patients. Figure 2 represents CTC images of an HNSCC patient where the first set of images is from a CTC cluster, and the second set is of individual CTCs from the same patient. Strong signals for Cytokeratin 18 (CK-18) and DAPI staining were observed in the detected CTCs. CK-18 provides mechanical support to the epithelial cell and is used as a marker for differentiating between various epithelial cells. The existence of CK-18 and the lack of a CD-45 signal prove that CTC originated from an epithelial lineage. Signals from DAPI staining indicated the CTC’s well-defined nucleus. The merged image was obtained from superimposed signals of DAPI, CD-45, and CK-18, where the CK-18 signal showed a homogenous distribution in CTC.

### Demographic and socioeconomic risk factors of selected CTC-based HNSCC patients

**Fig. 3:**
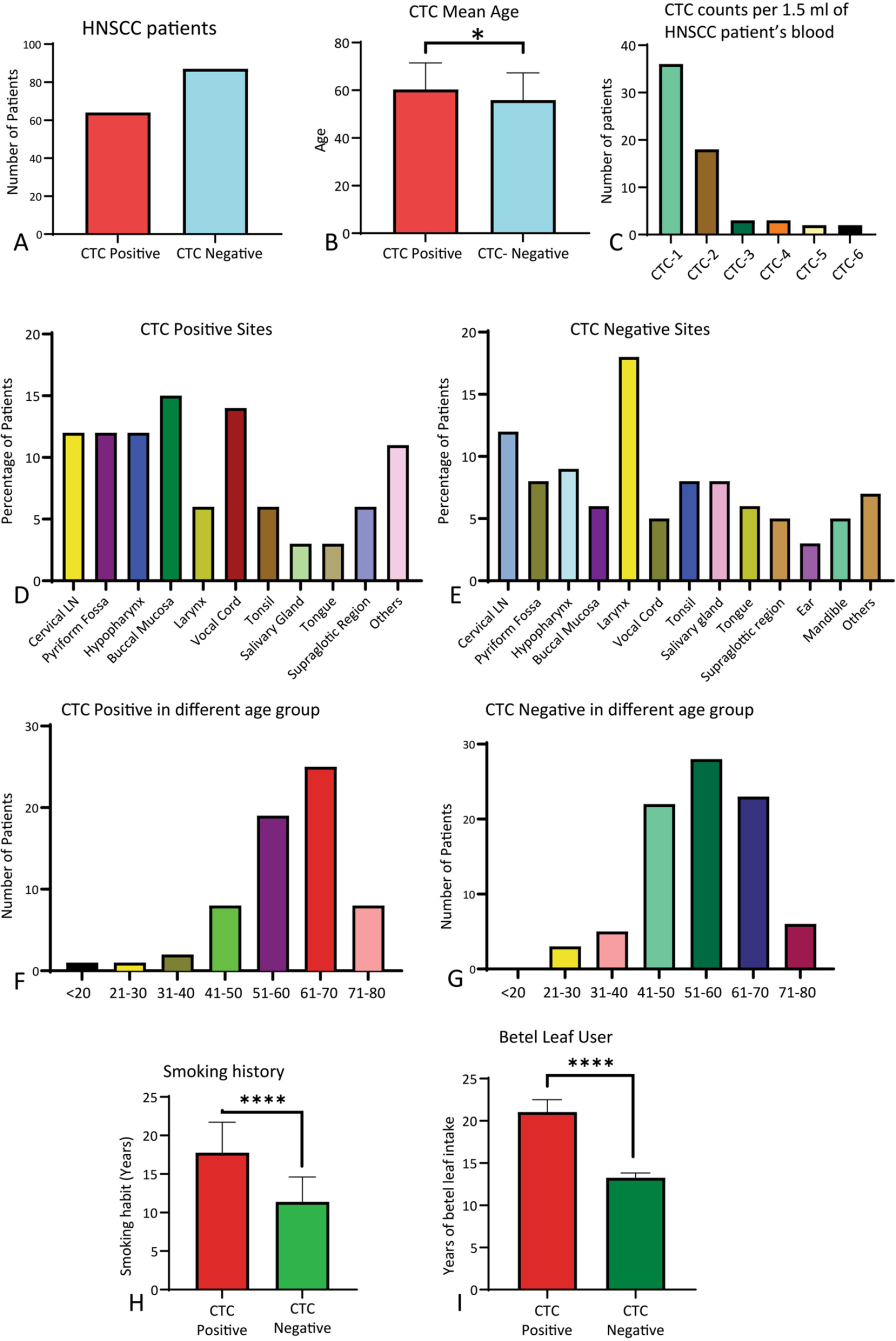
Demographic and socioeconomic risk factors considered to select CTC-based HNSCC patients. **A** HNSCC patients were categorized into two groups: CTC-positive (CTCs detected in blood) and CTC-negative patients (CTCs not detected in blood). **B** The mean age difference of CTC-positive and CTC-negative HNSCC patients. Data is represented here as mean ± SD. *P* < 0.05, a two-tailed unpaired t-test was carried out to compare the ages of CTC-positive and negative patients. **C** CTC count per 1.5 ml of blood in CTC-positive HNSCC patients. **D**, **E** Anatomical site distribution of CTC-positive and negative HNSCC patients. The number of HNSCC patients is presented as a percentage. **F**, **G** Distribution of CTC-positive and negative HNSCC patients based on age groups. The study subjects were grouped into different age groups with 10-year intervals. **H** Comparison of CTC-positive patients to CTC-negative patients based on the duration of smoking. Mann–Whitney unpaired two-tailed t-test was performed here as mean ± SD. *P* < 0.05 (**I**) Duration of Betel leaf use in CTC-positive and CTC-negative groups. Mann -Whitney unpaired two-tailed t-test was performed here as mean ± SD. *P* < 0.05. ****p-value < 0.0001

Demographic and socioeconomic parameters were considered to select the patient cohort for this study. Among the selected HNSCC patients, 64 individuals (42%) tested positive for CTCs, while the remaining 87 (58%) were CTC negative based on the presence of CTC in their blood (Fig. 3A). Therefore, we categorized our study subjects into two groups based on the presence of CTC cells (CTC-positive and CTC-negative). The mean age of the CTC-positive group was significantly higher (*p* < 0.05) compared to the CTC-negative group (Fig. 3B). Among the CTC-positive patients, 1 CTC cell was found in 36 HNSCC patients, 2 CTC cells in 18 patients, 3 CTC cells in 3 patients, 4 CTC cells in 3 patients, 5 CTC cells in 2 patients, and the highest 6 CTC cells observed in 2 HNSCC patients (Fig. 3C). Regarding the site of tumor occurrence, buccal mucosa and vocal cord showed a higher predisposition in CTC-positive patients, whereas larynx and cervical lymph node involvement were more prevalent in CTC-negative patients (Fig. 3D, E). CTC-positive patients were most prevalent in the 61–70 age group, while CTC-negative patients were more prevalent in the 51–60 age group (Fig. 3F, G). Moreover, 64% of the CTC-positive patients had a positive smoking history, which was statistically significant (*p* < 0.0001) compared to CTC-negative patients (Fig. 3H). Among the study patients, 45.7% of the HNSCC patients had a history of betel nut use, which was significantly higher (*p* < 0.0001) in the CTC positive group compared to the CTC negative group (Fig. 3I). Therefore, in Bangladesh, a history of chronic tobacco use (smoking and betel leaf consumption) is linked with the presence of CTCs in HNSCC patients.

### CTC- a biomarker for the diagnosis of early metastasis and therapeutic management

**Fig. 4:**
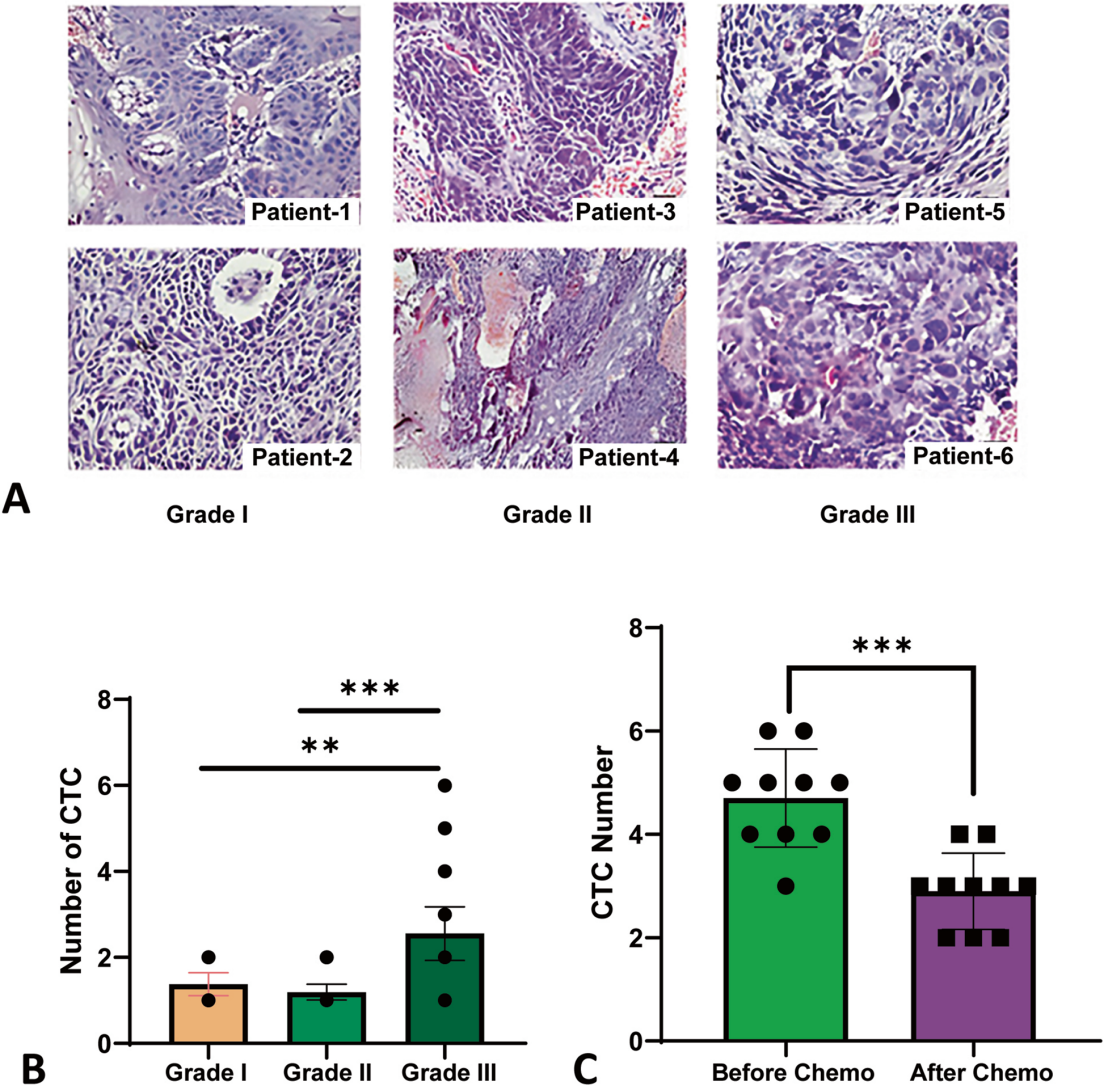
Histopathological observation and CTC count in pre/post-chemotherapy in CTC positive HNSCC (**A**) Histopathological representation of studied subjects based on Grade. Two images in the first column depict the image of two patients (Patients 1 and 2) of Grade I, the middle column represents the images of two patients (Patients 3 and 4) of Grade II, and the last column represents the images of two patients (Patients 5 and 6) of Grade-III. Histopathological slides were examined using the Hematoxylin and Eosin (H/E) procedure. **B** Distribution of CTC-positive patients based on the grades and CTCs count (*n* = 64). Black circular dots represent the number of CTCs (*n* = 64). Data is represented here as mean ± SD and one-way ANOVA was carried out to compare the number of CTCs and the Grade of CTC-positive patients and found highly significant. **C** The number of CTCs in blood samples was tested before and after three cycles of chemotherapy (*n* = 10). Mann–Whitney unpaired nonparametric unpaired t-test was performed and found highly significant. **p-value < 0.01, ***p-value < 0.005

Through microscopic examination, cancer grading determines whether cancer cells are regular or aberrant. The more aberrant the cells look, the higher the grade, and the faster the cancer is likely to spread. We graded the study patients according to histopathological findings to see the association of CTCs with cancer grade. The representative pictures of the tissue sections according to grade are displayed in Fig. 4A. Data reported that grade 3 had the highest number of CTC compared with grades 1 and 2 (Fig. 4B). Follow-up of the selected CTC-positive HNSCC patients (*n* = 10) for three cycles of chemotherapy, the CTC count was found to have significantly decreased (*p* < 0.0005) following treatment (Fig. 4C). Taken together, detecting CTCs may serve as a prospective prognostic marker for post-treatment surveillance and early metastasis diagnosis in HNSCC patients.

### Clinicopathological features of the study cohort

**Fig. 5:**
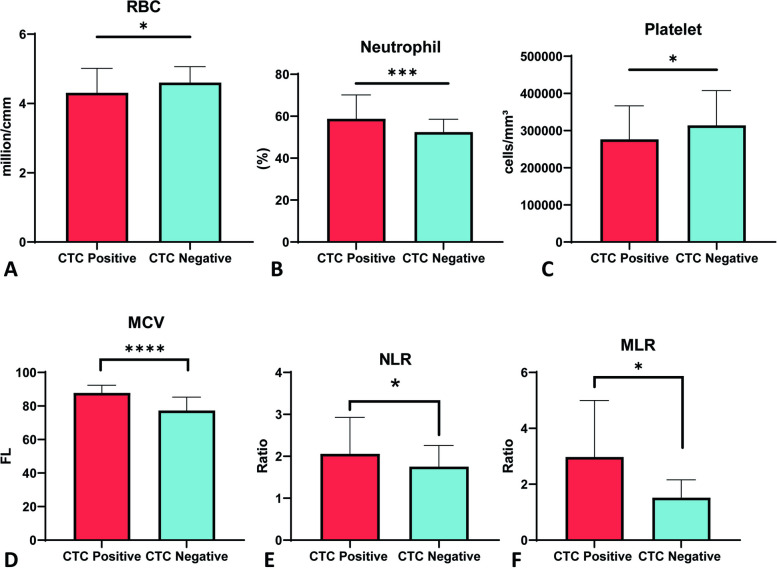
Enumeration of clinicopathological factors between CTC positive and CTC negative groups. **A** Red blood cell (RBC) was counted in million/cmm using the Neubauer Chamber method and compared between CTC-positive than CTC-negative patients. **B** Leishman staining was used to count neutrophils, and the results were compared between patients with CTC-positive and CTC-negative patients. **C** Platelet was counted using the Leishman staining method and compared between CTC-positive and CTC-negative patients. **D** The physiological protocol determined mean corpuscular volume (MCV) levels by dividing the RBC count by the hematocrit and multiplying the result by ten. **E**, **F** Neutrophil-to-lymphocyte ratio (NLR) and monocyte-to-lymphocyte ratio (MLR) levels were calculated manually. *p-value < 0.05, ***p-value < 0.005, ****p-value < 0.0001 two-tailed unpaired t-test

Hematological parameters are widely used to diagnose the disease severity and to compare disease and healthy conditions. In chronic diseases like cancer, these parameters often deviate from normal ranges. In this study, the mean red blood cell (RBC) count was found to be significantly decreased (*p* < 0.05) in the blood samples of CTC-positive patients compared to CTC-negative patients (Fig. 5A). CTC-positive patients had significantly higher neutrophil counts than CTC-negative patients (*p* < 0.005) (Fig. 5B). Platelet counts were significantly lower in CTC-positive groups (*p* < 0.05) (Fig. 5C). Moreover, CTC-positive patients had higher mean corpuscular volume (MCV) compared to CTC-negative patients (*p* < 0.0001) (Fig. 5D). The neutrophil–lymphocyte ratio (NLR) and monocyte-lymphocyte ratio (MLR) were also significantly higher in the CTC-positive group compared to the CTC-negative group (*p* < 0.05) (Fig. 5E, F ). No significant correlation was observed while multiple linear regression analysis was carried out using CTC as a dependent variable against different hematological parameters (Table 1). Besides these, no significant correlation was observed in white blood cell (WBC) count, lymphocyte count, eosinophil count, monocyte count, hemoglobin levels, hematocrit levels, mean corpuscular hemoglobin (MCH), serum creatinine levels, mean corpuscular hemoglobin concentration (MCHC), serum glutamic pyruvic transaminase (SGPT) levels, and erythrocyte sedimentation rate (ESR) values between the CTC-positive and CTC-negative groups (Supplementary Figs. 1, 2 and 3 and Supplementary Table 2).

**Table 1:** Multiple linear regression analysis using CTC as a dependent variable against different hematological parameters Coefficients^a^

*Parameters*	*Unstandardized coefficient*		*Standardized coefficient*	*t*	*Significance (P value)*	*95% CI for B*		Normality test^*^ (α = *0.05)*
	*B*	*Standard Error*	*Beta*			*Lower Bound*	*Upper Bound*	
Intercept	1.782	4.433		0.4020	0.6896	-7.146	10.71	Passed
WBC	-3.155e-005	0.0001044	0.1477	0.3022	0.7639	-0.0002418	0.0001787	Passed
Lymphocyte	0.01038	0.02813	0.2819	0.3688	0.7140	-0.04629	0.06704	Passed
Eosinophil	0.2761	0.1423	0.2121	1.940	0.0587	-0.01057	0.5627	Passed
Monocyte	0.06533	0.1223	0.2732	0.5344	0.5957	-0.1809	0.3116	Passed
Hematocrit	-0.01717	0.04031	0.3545	0.4259	0.6722	-0.09836	0.06402	Passed
Hemoglobin	0.04386	0.1716	0.1367	0.2556	0.7995	-0.3018	0.3895	Passed
MCH	0.02341	0.03223	0.3170	0.7262	0.4715	-0.04151	0.08833	Passed
S. Creatinine	-0.3047	1.114	0.1741	0.2735	0.7857	-2.548	1.939	Passed
MCHC	-0.06824	0.07537	0.3164	0.9053	0.3701	-0.2201	0.08357	Passed
SGPT	-0.02754	0.02009	0.2392	1.371	0.1772	-0.06800	0.01292	Passed
ESR	0.02154	0.01175	0.2417	1.833	0.0735	-0.002134	0.04521	Passed

More details on Supplementary Table 2

*CI* confidence interval

^*^Normality test was used using Kolmogorov–Smirnov (distance) test

^a^Dependable variable is the CTC value

### Shh and Nrf2 expression in HNSCC patients based on the presence of CTCs

**Fig. 6:**
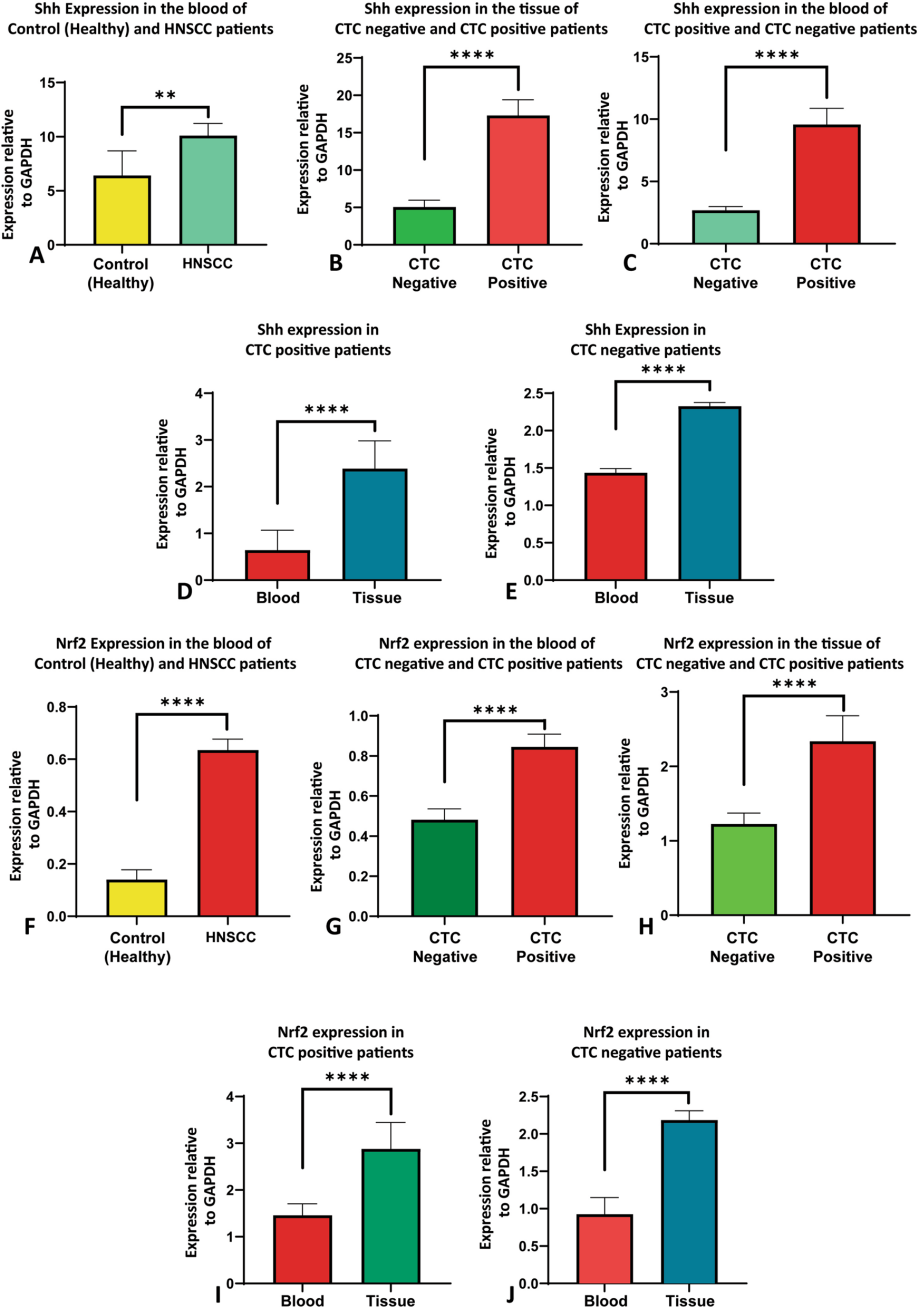
Expression of Shh and Nrf2 in blood and tissue samples based on CTCs. **A** Shh expression was quantified by qRT-PCR normalized against GAPDH in blood samples of HNSCC patients and compared with healthy control. **B**, **C** qRT-PCR was used to measure Shh expression in study subjects’ blood and tissue samples, normalized against GAPDH. The results were compared between patients who tested positive for CTC and those who did not. **D**, **E** qRT-PCR was used to quantify Shh expression in both CTC-positive and CTC-negative HNSCC patients. The results were normalized against GAPDH and compared between tissue and blood samples. **F** The expression of Nrf2 was measured in blood samples from study participants using qRT-PCR, normalized against GAPDH, and compared to healthy control subjects. **G**, **H** The expression of Nrf2 was also measured in study subjects’ blood and tissue samples using qRT-PCR, normalized against GAPDH, and compared between patients who tested positive for CTC and those who tested negative. **I**, **J** Quantitative real time PCR was carried out to determine Nrf2 expression in both blood and tissue samples from patients with HNSCC who were CTC-positive and negative, normalized against GAPDH. Data represented as mean ± SD and the Mann–Whitney test was carried out to compare Shh and Nrf2 expression among the study subjects. **p-value < 0.01, ****p-value < 0.0001, two-tailed unpaired t-test (Mann–Whitney test)

HNSCC patients who exhibit overexpression of Shh and Nrf2 have shown reduced disease-free survival (DFS) and overall survival (OS) [6]. Additionally, a higher number of circulating tumor cells (CTCs) has been associated with more aggressive HNSCC and poorer survival rates [17]. Therefore, the expression of Shh and Nrf2 was compared between CTC-positive and CTC-negative patient groups in blood and tissue samples. In the blood samples, Shh expression was found to be significantly higher in HNSCC patients compared to healthy controls (*p* < 0.01) (Fig. 6A). Shh expression was found to be significantly higher in CTC-positive patients compared to CTC-negative patients (*p* < 0.0001) (Fig. 6B). Furthermore, in the tumor tissue samples, Shh expression was significantly higher in CTC-positive patients compared to CTC-negative patients (*p* < 0.0001) (Fig. 6C). Moreover, Shh expression was significantly higher in tissue samples compared to blood samples in both the CTC-positive and CTC-negative groups of HNSCC patients (*p* < 0.0001) (Fig. 6D, E).

On the other hand, the expression of Nrf2 was found to be significantly higher in the blood samples of HNSCC patients compared to healthy controls (*p* < 0.0001) (Fig. 6F). The expression of Nrf2 was seen to be significantly higher in the blood samples of CTC-positive patients compared to CTC-negative patients (Fig. 6G). Furthermore, Nrf2 expression was significantly higher in the tumor tissue samples of CTC-positive patients compared to CTC-negative patients (*p* < 0.0001) (Fig. 6H). Additionally, Nrf2 expression was significantly higher in tissue compared to blood samples of the CTC-positive and CTC-negative groups of HNSCC patients (*p* < 0.0001) (Fig. 6I, J). Therefore, there is a correlation between Shh and Nrf2 expression and CTCs in HNSCC patients.

### Expression of CTC with Shh and Nrf2 expression in HNSCC during pre/post-treatment surveillance

**Fig. 7:**
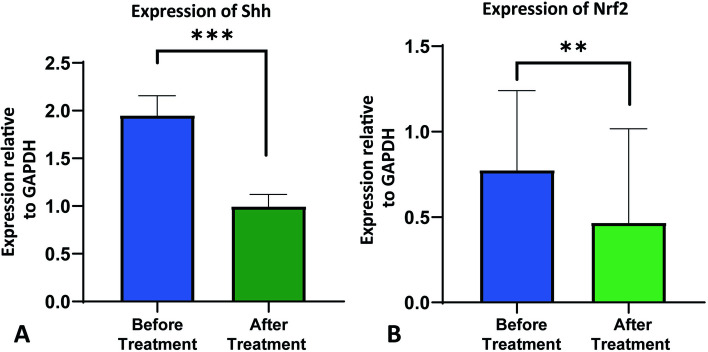
Expression of Shh and Nrf2 before and after chemo and/or radiotherapy (**A**) Quantitative real-time (qRT-PCR) was used to measure Shh expression in study participants’ blood samples before and after treatment, normalized against GAPDH. **B** Nrf2 expression was also quantified by qRT-PCR normalized against GAPDH in blood samples of study subjects at pre- and post-treatment phage. Data is represented here as mean ± SD, and the Mann–Whitney test was carried out to compare Shh and Nrf2 expression before and after treatment. * *p*-value < 0.05, *** *p*-value < 0.005, two-tailed unpaired t-test

Radiation therapy and/or chemotherapy with a regimen consisting of Cisplatin, 5FU, and Paclitaxel is the standard course of treatment for HNSCC in Bangladesh [19]. We further examined the blood samples of our patient cohort (*n* = 10) and grouped them into two groups, before treatment and after treatment, to determine whether the expression of Shh and Nrf2 changes following treatment. Our results showed that following chemotherapy and/or radiation therapy, there was a significant decrease in the expression of both Shh and Nrf2 (Fig. 7A, B).

### Association of CTCs in overall survival of HNSCC patients

**Fig. 8:**
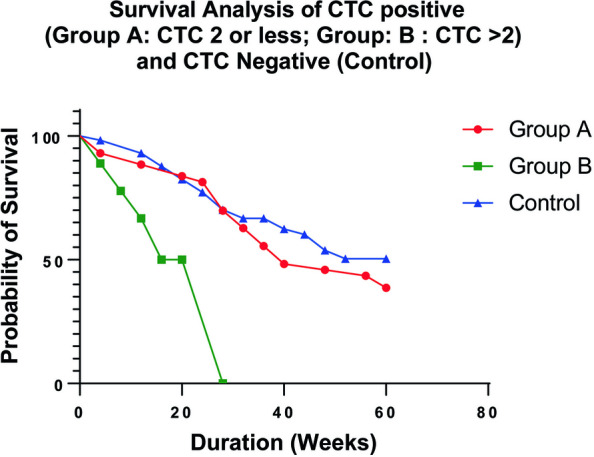
Survival analysis using Kaplan-Meyer (KM) plotter. Kaplan Meier analysis for overall survival in the patient cohort according to the number of CTCs to analyze the survival difference with statistical significance. We categorized CTC negative as control and CTC positive divided into two groups: namely group A (CTC ≤ 2) and group B (CTC > 2). * *p* < 0.05

Survival analysis is a widely used statistical technique in health research to analyze the time until an event of interest occurs. The Kaplan–Meier curve, a graphical representation of the probability of survival over time, is frequently used in cancer survival analysis. We categorized the CTC-positive patients as the test group and the CTC-negative patients as the control group. Next, we plotted the survival curves after stratifying the CTC-positive patients into two groups: Group A (CTC ≤ 2) and Group B (CTC number > 2). We performed a Log-rank (Mantel-Cox) test and found a statistically significant Chi-square value of 11.85 (df = 2) with a *p*-value of 0.0027. The median survival time differs between groups A (40) and B (22). The study’s survival analysis also revealed that, out of the HNSCC patients that were examined, 18 patients from Group A (CTC ≤ 2) and 15 patients from the CTC-negative group were at risk (Supplementary Table 3). Survival analysis of HNSCC patients with different CTC scores revealed that patients with a higher number of CTCs have poor overall survival in HNSCC (Fig. 8).

## Discussion

Head and neck squamous cell carcinoma (HNSCC) is a devastating and heterogeneous cancer [21]. Worldwide, HNSCC is the sixth deadliest cancer, with almost a million new cases detected every year and 50% mortality in low-income countries. The five-year overall survival of HNSCC is less than 50% [22], which warrants emphasis on early diagnosis along with monitoring of treatment outcomes. To address this concern, we aimed to develop novel diagnostic, prognostic, and therapeutic markers for head and neck squamous cell carcinoma. By using the TCGA dataset, we revealed that Shh (48%) and Nrf2 (34%) had been altered in HNSCC (Fig. 1). The alteration is higher in male compared to female patients (Fig. 1C, G). Alterations of Shh and Nrf2 in HNSCC patients were observed in all age groups, suggesting that Shh and Nrf2 expression is independent of age in HNSCC (Fig. 1D, H). Our data, too, has suggested a similar trend where both Shh and Nrf2 are overexpressed in HNSCC. We observed that males are more susceptible to HNSCC than females in both CTC-positive and CTC-negative cases (Fig. 3B). Canning M et al. reported that males are at greater risk for the development of HNSCC and the metastatic progression of HNSCC [23]. The mean age of CTC-positive patients is greater than that of CTC-negative patients (Fig. 3C). Only two individuals in our study had the highest number of CTCs (6 CTCs). In contrast, most patients had one or two CTCs (Fig. 3D). According to Yu et al., metastatic breast cancer patients had more than 5 CTCs per 7.5 mL of blood [24]. Also, higher CTC numbers correspond with aggressive disease, less time to relapse, and increased metastasis [25].

Circulating tumor cell (CTC) identification is a novel concept in diagnosing many cancers, including HNSCC at the early metastatic stage and surveillance of cancer treatment. It is a minimally invasive liquid biopsy technique to detect early metastasis and monitor treatment outcomes [26]. Our study found that the age range of 61–70 years was typical for CTC positivity, whereas the age range of 51–60 years was typical for CTC negativity (Fig. 3F, G). However, Wu et al. reported no significant correlation between CTCs, age, and gender in HNSCC patients [27].

Smoking is directly associated with the development of HNSCC, including a more extended period of smoking [28], and the use of betel leaf is a self-reliant risk factor for the occurrence of HNSCC [29]-the use of tobacco accounts for 1.6 million deaths each year in Southeast Asia. Approximately 10–20% of the world’s population consumes betel quid and tobacco; approximately 81% of this population is from Southeast Asian Regions, including Bangladesh. Our study tested whether using these two tobacco products is associated with detecting CTCs. Our data demonstrated that HNSCC patients having a history of smoking and betel nut consumption correlated with the presence of CTC (Fig. 3H, I). A similar result was observed in a study where they reported that smoking alone is an independent important risk factor for the genesis of HNSCC [30]. Another study also reported that betel nut chewing history is a significant risk factor for locally advanced HNSCC [31]. Therefore, smoking, and betel nut use are independent risk factors for the detection of CTCs in HNSCC.

The number of CTCs was correlated with the advancing clinical stage of the disease in many epithelial-originated cancers, including HNSCC. Buglione et al. showed that stage IV patients had higher CTCs than stage I-III [32]. Our data demonstrated that Grade-3 HNSCC patients exhibit the highest number of CTCs compared to grade 1 and grade 2 (Fig. 4B). A study showed that the presence of CTCs is only correlated to the T and M stages [33], while another study concluded that CTC counting is related to the N stage rather than the T or M stage [34]. Hence, it is evident that the number of CTCs is increasing in higher-grade HNSCC patients compared to low grade. Identification of CTCs may aid in evaluating the response to cancer treatments. Our result has shown that the number of CTCs decreased significantly after three cycles of chemotherapy (Fig. 4C). McMullen et al. also reported a similar finding in HNSCC patients, mentioning CTC as a potential maker for treatment response in HNSCC [35].

Next, in our study, we also analyzed the clinicopathological features of our subjects based on their CTC count. In our recent clinical trial in 152 oral cancer patients, we observed that the preoperative CTC levels showed a stronger correlation with adverse clinicopathological factors. The outcome suggested the role of CTCs as a sensitive prognostic marker to predict survival outcomes and disease progress [20]

We tested multiple hematological parameters of CTC-positive and negative HNSCC patients. Anemia is a prevalent physiological feature in all cancers, including HNSCC [36]. Our study showed a significant reduction in RBC count in CTC-positive patients compared to CTC-negative patients (Fig. 5A). The neutrophil count in patients positive for CTC was significantly higher than in negative patients (Fig. 5B). There was a significant decrease in platelet count in CTC-positive patients compared to CTC-negative patients (Fig. 5C). Szczerba et al. reported a significant association between CTCs and neutrophils in breast cancer patients and animal models [37]. This observation supports our findings that there is an association between CTCs and neutrophil levels in HNSCC [38]. In a recent study, it was observed that there is a positive correlation between platelet count and CTC count in prostate cancer. The same study also reported that the total lymphocyte count was positively correlated with the CTC count in prostate cancer [39]. MCV itself is correlated with the survival of HNSCC patients [40]. There was a significant difference in MCV levels between CTC-positive and CTC-negative groups (Fig. 5D). The neutrophil–lymphocyte ratio (NLR) is one of the significant predictors in many cancers, including HNSCC [41]. Our study showed a significant relation of NLR in both CTC positive and CTC negative patients, which may be due to both groups being from HNSCC (Fig. 5E). Monocyte-lymphocyte ratio (MLR) in the peripheral blood of HNSCC also serves as a worse prognostic marker [42]. Our study observed significant MLR in both CTC-positive and CTC-negative patients (Fig. 5F). For the first time, we have shown the difference in multiple hematological parameters based on the presence or absence of CTCs in blood in HNSCC patients.

One of our main objectives was to investigate and compare Shh and Nrf2 expression in HNSCC patients’ tumor tissue based on the presence or absence of CTCs. Shh and Nrf2 were reported to be overexpressed in HNSCC patients [43, 44]. CTC is a novel concept in the early diagnosis of HNSCC to extend the survival rate of HNSCC patients. We reported the novel correlation between Shh and Nrf2 expression with CTCs in HNSCC. Diagnostically and prognostically essential markers such as Shh and Nrf2 are not investigated in the context of CTCs in HNSCC. Previously, our group showed that Shh and Nrf2 are potential therapeutic and prognostic biomarkers for HNSCC [6]. Therefore, we planned to investigate the association between the expression of Shh/Nrf2 and the presence of CTCs to establish these biomarkers as prognostic markers for early diagnosis and therapeutic management in HNSCC.

Sonic hedgehog is overexpressed in many cancers, including HNSCC [45]. In our study, we found that Shh was overexpressed in the blood of HNSCC (both CTC positive and negative patients) compared to controls (healthy volunteers) (Fig. 6A). Shh overexpressed in the blood of CTC-positive patients compared to that of CTC-negative patients (Fig. 6B). Shh was also shown to be significantly overexpressed in the tissue of patients who had CTCs as opposed to those who did not (Fig. 6C). CTC-positive and CTC-negative HNSCC patient groups showed considerably increased Shh expression in tissue samples compared to blood (Fig. 6D, E). In our study, Nrf2 was also observed to be overexpressed in HNSCC (both CTC-positive and CTC-negative) patient blood compared to healthy subjects (Fig. 6F). Overexpression of Nrf2 was observed in CTC-positive patient’s blood compared with CTC-negative patients’ blood (Fig. 6G). Nrf2 was also shown to be significantly overexpressed in the tissue of patients who had CTCs as opposed to those who did not (Fig. 6H). CTC-positive and CTC-negative HNSCC patient groups showed considerably increased Nrf2 expression in tissue samples compared to blood (Fig. 6I, J).

We further analyzed our patient cohort and categorized it into two groups before and after treatment. We observed that Shh and Nrf2 expression significantly decreased after chemotherapy and or radiotherapy (Fig. 7A, B). The Kaplan-Meyer survival analysis curve showed a poor survival outcome for patients with higher CTC (Fig. 8). According to Kim H et al., overall survival (OS) was inversely correlated with the total number of CTCs following chemotherapy, and a lower number of CTCs after chemotherapy was substantially linked to a longer OS in pancreatic ductal adenocarcinoma [46]. The sample sizes for men and women in this study were not comparable. The sample sizes for the high and low grades were also unrelated. It would be rational if only one histopathologist rated the HNSCC patients rather than several pathologists.

## Conclusions

Shh and Nrf2 were highly expressed in HNSCC patients’ blood and tumor tissue samples compared to CTC-negative HNSCC patients. Shh and Nrf2 were overexpressed in the tumor tissue of both CTC-positive and CTC-negative patients compared to blood. Smoking and betel leaf users were at risk of having CTC in HNSCC patients, and non-smokers and non-chewers had no CTC or fewer CTCs. In CTC-positive patients compared to CTC-negative patients, RBC and Platelet counts were dramatically decreased, although Neutrophil count, MCV, NLR, and MLR were significantly increased. In our study subjects, buccal mucosa and vocal cord are common sites of cancer occurrence in CTC-positive patients. Ages 61 to 70 are more susceptible to CTC positivity. In HNSCC patients who tested positive for CTCs following treatment, the frequency of CTCs considerably decreased. It was also observed that Shh and Nrf2 expression was decreased in post-treatment (combination regimen of Cisplatin, 5FU, and Paclitaxel). In conclusion, CTC detection, Shh, and Nrf2 overexpression could be ideal biomarkers for prognostic, therapeutic, and diagnostic purposes in HNSCC.

### Supplementary Information

**Additional file 1.**

**Additional file 2.**

**Additional file 3.**

**Additional file 4: S1 Table.** List of primers used for qRT-PCR.

**Additional file 5: S2 Table.** Summary of hematological investigations.

**Additional file 6: S3 Table.** Patients at risk.

## Data Availability

No datasets were generated during the current study. Data will be available on request.

## Abbreviations

Anti-EpCAM: Anti Epithelial Cell Adhesion Molecule Antibody
CBC: Complete blood count
cDNA: Complementary DNA
CTC: Circulating Tumor Cell
DAPI: 4′,6-Diamidino-2-phenylindole
DFS: Disease-free survival
ESR: Erythrocyte sedimentation rate
ENT: Otolaryngology and Head Neck Surgery
5-FU: 5-Fluro uracil
GAPDH: Glyceraldehyde 3-phosphate dehydrogenase
GLI1: GLI Family Zinc Finger 1
HNSCC: Head and Neck Squamous Cell Carcinoma
Keap1: Kelch-like ECH-associated protein 1
MCH: Mean corpuscular hemoglobin
MCHC: Mean corpuscular hemoglobin concentration
MCV: Mean corpuscular volume
MLR: Monocyte-to-lymphocyte ratio
MYC: Master Regulator of Cell Cycle Entry
Nrf2: Nuclear factor erythroid 2-related factor 2
NLR: Neutrophil-to-lymphocyte ratio
OS: Overall Survival
PTCH1: Protein patched homolog 1
PCR: Polymerase chain reaction
qRT-PCR: Quantitative real time PCR
RBC: Red blood cell
SGPT: Serum Glutamic Pyruvic Transaminase
Shh: Sonic Hedgehog (Shh)
SMO: Smoothened
ssDNA: Single-stranded DNA
SUFU: Suppressor of fused homolog
TCGA: The Cancer Genome Atlas
TPM: Transcript per millionca
WBC: White blood cell
