# Supplementary figures and images for: CTC together with Shh and Nrf2 are prospective diagnostic markers for HNSCC
Source: BMC Mol Cell Biol. 2024 Feb 10;25:4. doi: 10.1186/s12860-024-00500-0 (PMC10858504; doi:10.1186/s12860-024-00500-0)

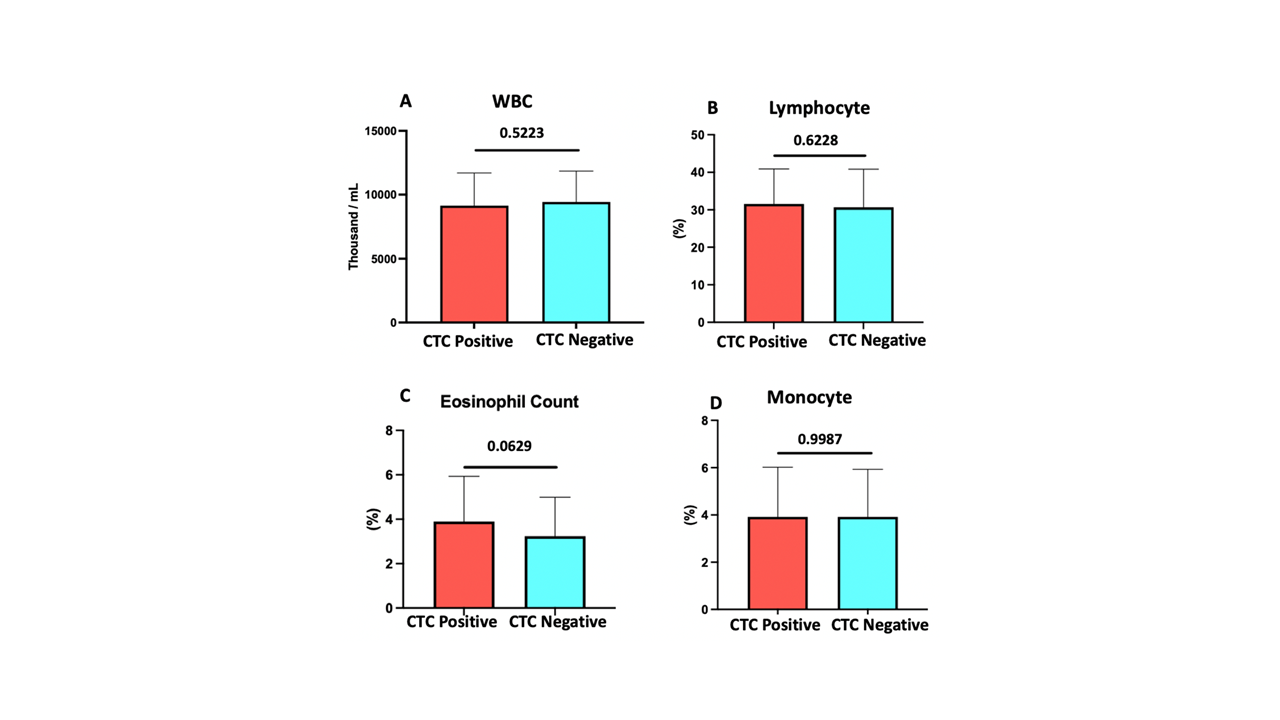

Supplement: Supplementary file 1 — Additional file 1. [file 12860_2024_500_MOESM1_ESM.tif]

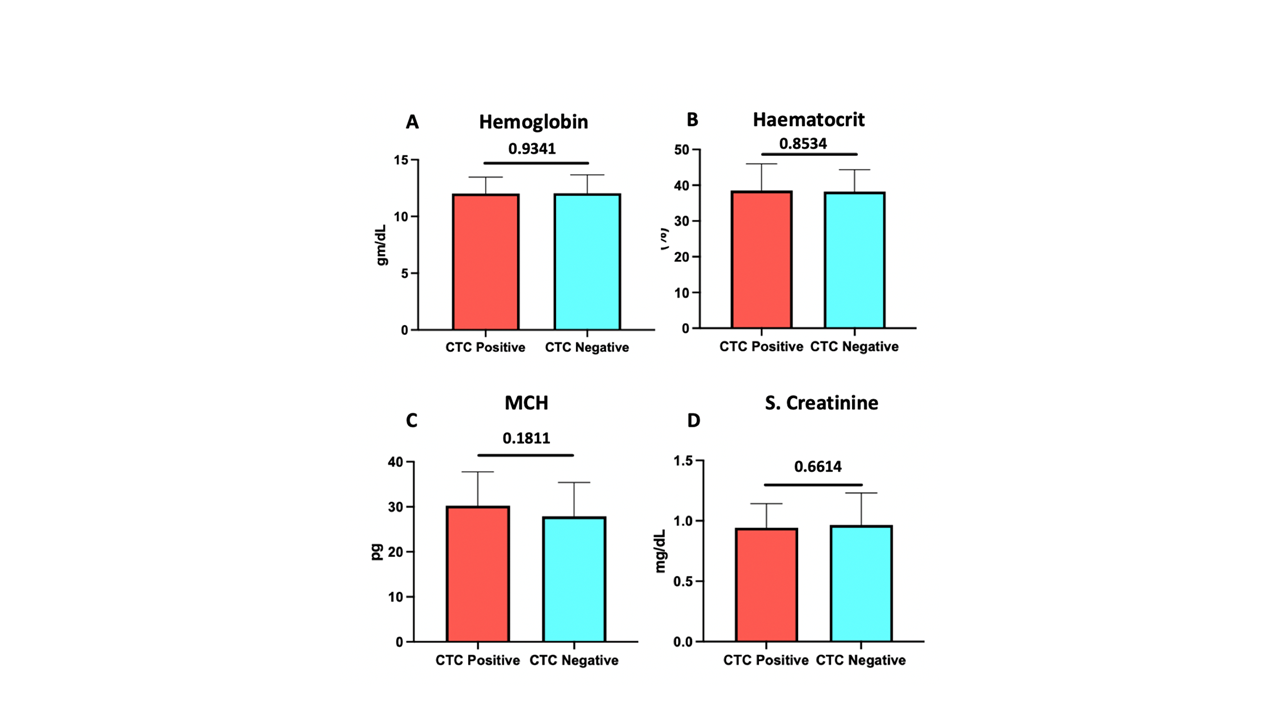

Supplement: Supplementary file 2 — Additional file 2. [file 12860_2024_500_MOESM2_ESM.tif]

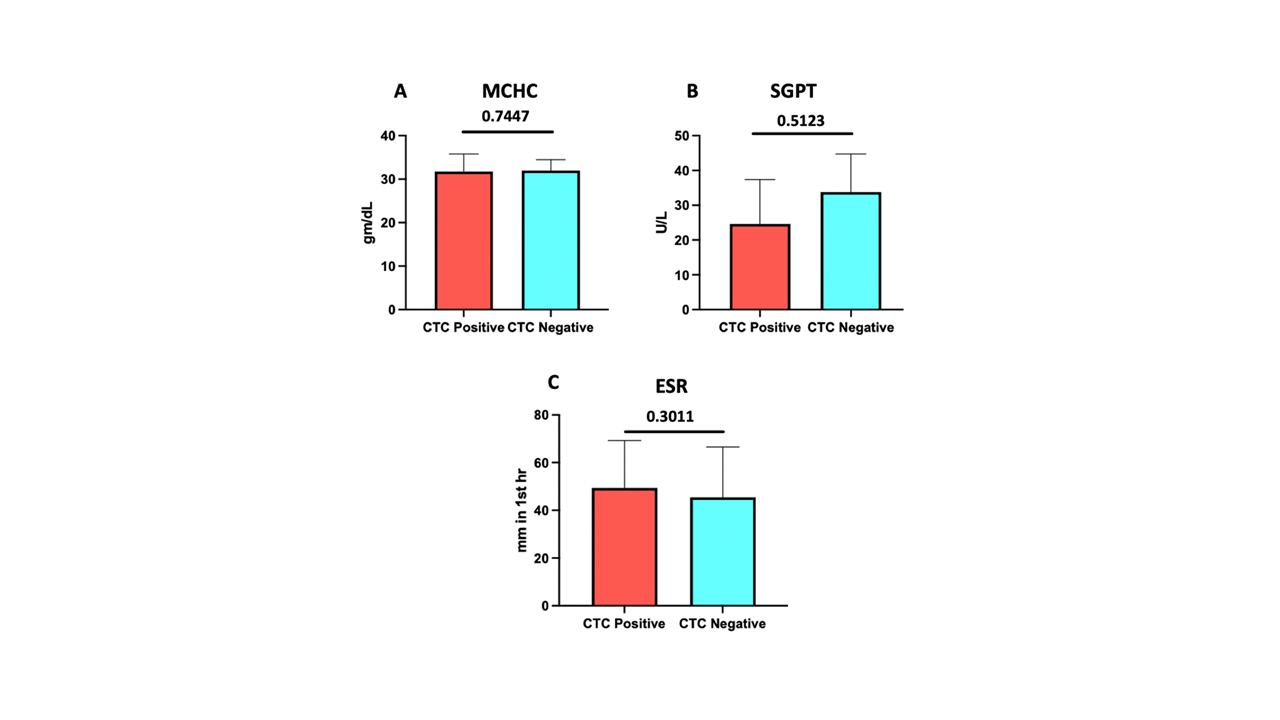

Supplement: Supplementary file 3 — Additional file 3. [file 12860_2024_500_MOESM3_ESM.tif]
