# Supplementary material for: CTC together with Shh and Nrf2 are prospective diagnostic markers for HNSCC
Source: BMC Mol Cell Biol. 2024 Feb 10;25:4. doi: 10.1186/s12860-024-00500-0 (PMC10858504; doi:10.1186/s12860-024-00500-0)
Supplement: Supplementary file 4 — Additional file 4: S1 Table. List of primers used for qRT-PCR. [file 12860_2024_500_MOESM4_ESM.pdf]

S1 Table: List of primers used for qRT-PCR

**Name of Primer**	**Direction**	**Sequence**	**Tm**	**Source**
**Shh** (Primer of targeted	F (5´→3´)	AGGACCCGGTTTGATCTTCT	56.5	**IDT**
	R (5´→3´)	GCCATGTFACACAGACAACC	57.4	
**Nrf2** (Primer of targeted gene)	F (5´→3´)	GCGACGGAAAGAGTATGAGC	55.7	
	R (5´→3´)	GTTGGCAGATCCACTGGTTT	55.6	
**GAPDH** (Primer of housekeeping	F (5´→3´)	CAGCCTCAAGATCATCAGCA	54.8	
	R (5´→3´)	TGTGGTCATGAGTCCTTCCA	55.7	
