# Supplementary material for: CTC together with Shh and Nrf2 are prospective diagnostic markers for HNSCC
Source: BMC Mol Cell Biol. 2024 Feb 10;25:4. doi: 10.1186/s12860-024-00500-0 (PMC10858504; doi:10.1186/s12860-024-00500-0)
Supplement: Supplementary file 5 — Additional file 5: S2 Table. Summary of hematological investigations. [file 12860_2024_500_MOESM5_ESM.pdf]

S2 Table

Summary of hematological investigations

Parameters	Unit	Normal Level	mean±sd		p-value	Statistical tool
			**CTC Positive**	**CTC Negative**		
**RBC**	Million/mL	4.7-6.1	4.306±0.7083	4.603±0.4569	**0.0280***	**Student's t test**
**WBC**	/mL	4500-11000	9142±2551	9430±2407	0.5223	**Student's t test**
**Neutrophil**	%	45-70	58.77±11.41	52.44±6.159	**0.0002***	**Student's t test**
**Lymphocyte**	%	18-45	31.57±9.292	30.67±10.13	0.6228	**Student's t test**
**Eosinophil**	%	2-6	5.604±7.163	4.056±4.349	0.1453	**Student's t test**
**Monocyte**	%	2-8	3.915±2.104	3.914±2.013	0.9987	**Student's t test**
**Platelet**	/Litre	150000-450000	276419±90288	314000±93792	**0.0466***	**Student's t test**
**Hemoglobin**	gm/dL	M:13.2-16.6 F:11.6-15	12.03±1.437	12.05±1.610	0.9341	**Student's t test**
**ESR**	mm in 1st hour	<15	43.27±24.96	40.18±26.69	0.5166	**Student's t test**
**S. Creatinine**	mg/dL	0.74-1.35	0.9433±0.1984	0.9653±0.2648	0.6614	**Student's t test**
**SGPT**	U/L	7-56	24.64±12.75	33.07±37.04	0.2592	**Student's t test**
**Hematocrit**	%	34.3-48.6	38.53±7.431	38.23±6.119	0.8534	**Student's t test**
**MCV**	fL	80-100	87.80±4.591	77.25±8.003	**<0001***	**Student's t test**
**MCH**	pg	27.5-33.2	32.20±13.55	27.89±7.514	0.0802	**Student's t test**
**MCHC**	gm/dL	32-36	31.75±4.018	31.99±2.474	0.7447	**Student's t test**
**NLR**			2.05672	1.75212	**0.0219***	**Student's t test**
**MLR**			2.97493	1.51821	**0.0100***	**Student's t test**
