# Supplementary material for: CTC together with Shh and Nrf2 are prospective diagnostic markers for HNSCC
Source: BMC Mol Cell Biol. 2024 Feb 10;25:4. doi: 10.1186/s12860-024-00500-0 (PMC10858504; doi:10.1186/s12860-024-00500-0)
Supplement: Supplementary file 6 — Additional file 6: S3 Table. Patients at risk. [file 12860_2024_500_MOESM6_ESM.pdf]

S3 Table
Patients at risk

Weeks elapsed	CTC <2	CTC>2	CTC Negative
0.000	43	9	57
4.000	43	9	57
8.000		8	
12.000	40	7	56
16.000		4	53
20.000	38	2	50
24.000	36		47
28.000	35	1	44
32.000	30		40
36.000	26		38
40.000	23		31
48.000	20		16
52.000			16
56.000	19		
60.000	18		15

After the period of study, 18 and 15 patients are at risk among the CTC positive and CTC negative patients respectively.
